# Exploring variation in the use of feedback from national clinical audits: a realist investigation

**DOI:** 10.1186/s12913-020-05661-0

**Published:** 2020-09-11

**Authors:** Natasha Alvarado, Lynn McVey, Joanne Greenhalgh, Dawn Dowding, Mamas Mamas, Chris Gale, Patrick Doherty, Rebecca Randell

**Affiliations:** 1 School of Healthcare and the Wolfson Centre for Applied Health Research, University of Leeds, Leeds, England; 2 School of Sociology and Social Policy, University of Leeds, Leeds, England; 3 School of Health Sciences, University of Manchester, Manchester, England; 4 Primary Care and Health Sciences, Keele University, Keele, England; 5 University of Leeds, Leeds, England; 6 Department of Health Sciences, York University, York, England; 7 Faculty of Health Studies and the Wolfson Centre for Applied Health Research University of Bradford, Bradford, England

**Keywords:** Audit and feedback, Realist evaluation, Programme theory, Quality improvement

## Abstract

**Background:**

National Clinical Audits (NCAs) are a well-established quality improvement strategy used in healthcare settings. Significant resources, including clinicians’ time, are invested in participating in NCAs, yet there is variation in the extent to which the resulting feedback stimulates quality improvement. The aim of this study was to explore the reasons behind this variation.

**Methods:**

We used realist evaluation to interrogate how context shapes the mechanisms through which NCAs work (or not) to stimulate quality improvement. Fifty-four interviews were conducted with doctors, nurses, audit clerks and other staff working with NCAs across five healthcare providers in England. In line with realist principles we scrutinised the data to identify how and why providers responded to NCA feedback (mechanisms), the circumstances that supported or constrained provider responses (context), and what happened as a result of the interactions between mechanisms and context (outcomes). We summarised our findings as Context+Mechanism = Outcome configurations.

**Results:**

We identified five mechanisms that explained provider interactions with NCA feedback: reputation, professionalism, competition, incentives, and professional development. Professionalism and incentives underpinned most frequent interaction with feedback, providing opportunities to stimulate quality improvement. Feedback was used routinely in these ways where it was generated from data stored in local databases before upload to NCA suppliers. Local databases enabled staff to access data easily, customise feedback and, importantly, the data were trusted as accurate, due to the skills and experience of staff supporting audit participation. Feedback produced by NCA suppliers, which included national comparator data, was used in a more limited capacity across providers. Challenges accessing supplier data in a timely way and concerns about the quality of data submitted across providers were reported to constrain use of this mode of feedback.

**Conclusion:**

The findings suggest that there are a number of mechanisms that underpin healthcare providers’ interactions with NCA feedback. However, there is variation in the mode, frequency and impact of these interactions. Feedback was used most routinely, providing opportunities to stimulate quality improvement, within clinical services resourced to collect accurate data and to maintain local databases from which feedback could be customised for the needs of the service.

## Background

Understanding if, how and why quality improvement strategies work in healthcare settings has been a research priority internationally for several decades [1]. In the United Kingdom’s (UK) National Health Service (NHS) such strategies include audit and feedback, a type of which are National Clinical Audits (NCAs). NCAs intend to stimulate quality improvement by systematically measuring care quality for different clinical specialities and patient groups [2]. They are well established in the NHS; first introduced in the 1990s, there are now over 50 that are either managed centrally by the Healthcare Quality Improvement Programme (HQIP), or by independent organisations [2, 3].

Significant resources, including clinicians’ time, are invested in collecting and submitting data to central databases for participation in NCAs. NCA suppliers manage these databases and produce feedback for local services to stimulate quality improvement. Key attributes of this feedback are that it has (or is at least intended to have) national coverage, it measures practice against clinical guidelines/standards and/or measures patient outcomes, and it monitors performance in an on-going way [4]. The introduction of NCAs has been followed by improvements in patient care in a number of clinical specialities, including hip fracture and acute coronary syndrome [5, 6]. However, there is variation within and across healthcare providers in the extent to which they engage with NCA feedback [3, 4], which indicates that it is not being used to its full potential.

Reported constraints on the use of NCA feedback include variable data quality or relevance, whilst supports include the credibility of NCA managing organisations and the use of multiple approaches in data interpretation [3, 7]. However, NCAs operate alongside and can embody other improvement strategies. For example, NCA performance measures can be linked to payment systems designed to incentivise high quality care, such as Best Practice Tariffs (BPT), and outcomes can be reported publically [8]. Further to this, the NHS is a complex and dynamic system, comprising multiple services and professional groups that interact with and adapt to improvement strategies when they are introduced into the system [9]. Therefore, explaining how NCAs work (or not) to stimulate quality improvement is challenging.

To better understand how NCAs and quality improvement strategies work, there are increasing calls for research that interrogates the theoretical assumptions that underpin their use [10–12]. Therefore, this study applied the principles of realist evaluation, a programme theory approach, to help explain variation in the use and impact of NCA feedback [13, 14]. The specific research questions addressed were how, why and in what contexts is NCA feedback used to stimulate quality improvement?

### Research strategy

**Fig. 1:**
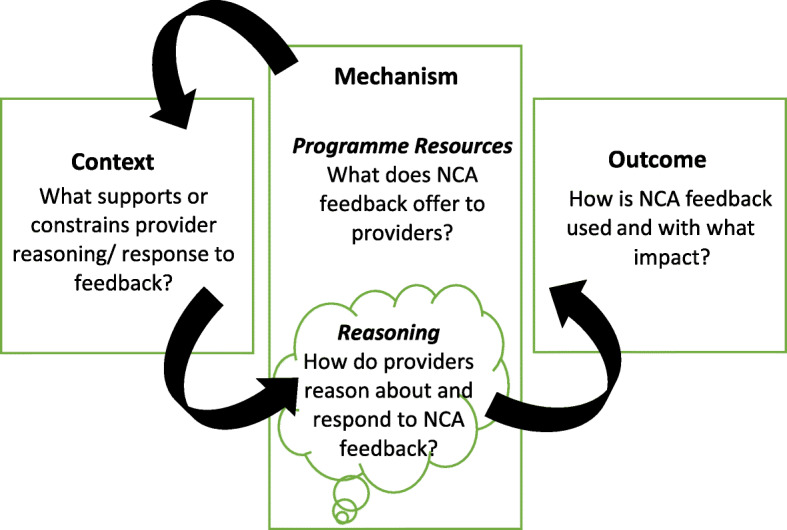
Context, Mechanism, Outcome configuration adapted from Dalkin et al. [16]

Realist evaluation examines how context shapes the mechanisms through which programmes/interventions work (or not) to produce outcomes. This examination is focused on constructing, testing and refining programme theory, configured as Context + Mechanism = Outcome (CMO). Figure 1 helps to define the CMO concepts and, specifically, how they were applied in this study using NCA feedback as an example programme. Put simply, CMOs are conceptual tools that help explore how interventions work in complex systems, leading to explanations of what about an intervention might work, for whom, how, why and in what circumstances [14, 15].

As shown in Fig. 1 [16], in this study mechanisms consist of what NCA feedback offers to services and how and why individuals/groups respond to it. Context refers to what, in the existing system into which the feedback is introduced, supports or constrains these responses, and can include cultural and structural influences, as well as personal attitudes and beliefs [17]. In terms of outcomes, of particular interest here was where interactions between healthcare providers and NCA feedback led to attempts to change practice to improve performance. However, understanding the success or failure of any change effort was beyond the remit of this study; our aim was to construct tentative programme theories to provide insight into why NCA feedback is used to stimulate quality improvement in some contexts but not in others. These tentative programme theories will require iterative testing for refinement as part of the evaluation cycle [18].

### Sampling strategy

To develop a programme theory applicable beyond a single NCA, the sampling strategy was designed to capture variation in three areas: (1) NCAs, (2) healthcare providers, and (3) healthcare professionals. The variation captured in these areas is described below.

#### NCAs

The NCAs sampled were the Myocardial Ischaemia National Audit Project (MINAP) and the Paediatric Intensive Care Audit Network (PICANet). These audits were chosen based on variation in clinical speciality, patient groups, and performance measures. MINAP includes a performance measure that is part of the BPT, whilst PICANET includes measures that are part of Commissioning for Quality and Innovation (CQUINS), which make a proportion of providers’ income conditional on demonstrating improvements in specified areas of patient care. Both PICANet and MINAP are managed centrally by HQIP. Therefore, to capture further variation in NCAs, we included professionals with experience of the National Audit of Cardiac Rehabilitation (NACR) and the British Association of Urological Surgeons (BAUS) audits, which are managed independently. See Table 1 below for summary of the variations captured.

**Table 1:** NCAs Sampled

NCA	Date initiated	Clinical speciality	Examples of NCA performance measures
PICANet	2002	Paediatric Intensive Care	• Standardised Mortality Ratio (SMR): ratio between the observed number of deaths and the number of deaths that would be expected, given the severity of patients’ illness at admission • Unplanned extubations - accidental removal of breathing tubes • Emergency readmissions within 48 h of discharge
MINAP	1998	Cardiology – Myocardial Infarctions (heart attacks)	• Call-to-Balloon time: time between ambulance call and Primary PCI treatment: target 90 min • Door-to-Angiography time: time between arrival at hospital to diagnostic procedure: target 72 h • Discharge on gold standard drugs: Proportion of patients who received all secondary prevention medication for which they were eligible
BAUS	2012–2016^a^	Urology - diseases of the urinary-tract system and the male reproductive organs	Cystectomy (bladder removal surgery) outcomes data, including: • 30 day mortality rate • 90 day mortality rate • length of hospital stay
NACR	2005	Cardiology - Cardiac Rehabilitation	• Cardiac Rehabilitation offered to all priority groups • Percent of patients with recorded assessment before starting formal Cardiac Rehabilitation: >England 80%; Northern Ireland 88%; Wales 68% • Percent of patients with recorded assessment at the end of Cardiac Rehabilitation: > England 57%, Northern Ireland 61%, Wales 43%

^a^Refers to first publication on BAUS website, data collection may have begun earlier

#### Healthcare provider

The NCAs were explored within five NHS Trusts (providers) in England that differed in size (populations served and staff numbers) and services offered. Three of the Trusts were large acute Teaching Hospitals serving populations of over 1 million and offering specialist services that included Paediatric Intensive Care Units (PICUs). The other two were smaller District General Hospitals (DGHs), serving populations of less than 1 million and without PICUs. Therefore, use of PICANet was investigated in PICUs across three Trusts and MINAP within cardiology services across all five.

#### Healthcare professional

Within each Trust, a combination of purposive and snowball sampling was used to recruit healthcare professionals [19]. The local collaborator for the study (typically a MINAP or PICANet lead) was interviewed first. These contacts were then asked to identify others who were involved with the NCAs targeted, such as those responsible for data collection, generating NCA outputs and/or using those outputs for quality monitoring and improvement. In this way, we were able to map the networks through which feedback is produced by certain stakeholders and used by others.

Some interview participants worked within professional groups that had oversight for quality monitoring across clinical services within their Trust, for example, some were members of the Boards that govern Trusts and Board sub-committees with a remit for quality and safety. In contrast to staff within clinical services, who tended to talk about their work with specific audits, these participants discussed their interaction with multiple NCAs in more general terms.

### Recruitment

The researchers contacted all individuals, who had agreed to have their contact details provided to the researchers as part of the snowball strategy described above, via email. If no response was received one follow-up email was sent; individuals who responded were sent a study information sheet and, if interested in participating, a time and date for the interview was arranged. Interviews took place at the individual’s place of work or via telephone if that was more convenient for them. Interviews were audio-recorded and informed consent was taken on the day of interview.

## Methods

The construction of CMO configurations can draw on documented (explicit) theory, as well as implicit theories that are ‘*personal constructions about particular phenomena[ …*] *which reside in individuals’ minds*’ (ICEBeRG [20]). In this study, the CMO configurations constructed were rooted in healthcare professionals’ experiences of using NCA feedback. Semi-structured interviews were conducted with these staff using realist ‘theory gleaning’ principles, whereby participants are asked to articulate how their contextual circumstances impact on their behaviour [18]. An interview schedule was developed that guided discussion of the participant’s role, with a focus on their responsibilities for NCAs e.g. data collection, review of data reports, and/or dissemination, how NCA data was collected, stored and managed, how feedback generated from NCA data was used e.g. in what format, in what routines. Participants were asked specifically, if and how NCA feedback were used to stimulate quality improvement and what challenges they experienced (constraints on use of NCA feedback).

### Data analysis

The interviews were transcribed verbatim and anonymised. A framework approach was applied in analysis, whereby a thematic framework was developed to categorise (code) the data and applied to transcripts in NVivo (qualitative data analysis software) [21]. Four researchers independently read the first three interview transcripts to identify themes in the data and came together to develop a tentative thematic framework. Themes and sub-themes were incorporated into the framework that reflected the realist concepts of context (operationalised as the characteristics of the organisational personnel, culture or infrastructure that appeared to support or constrain use of NCA data), mechanism (operationalised as the ways in which people responded to NCA feedback and why), and outcome (operationalised as the impact of these responses, with a focus on service changes intended to improve performance). The framework was entered innto NVivo, tested using five new transcripts and refined as necessary before being applied systematically to all transcripts. .

The CMO configuration is the main structure for analysis in realist evaluation [22]. To construct CMOs, the researchers explored the coded data to identify instances where NCA feedback was reported to have been used by providers. We were particularly interested in instances where use of feedback had stimulated quality improvement in the form of practice changes, but also sought to capture other endpoints, such as where feedback was used for assurance (confirming performance complies with certain standards). The data were then interrogated to understand how and why feedback was used in these ways, and what supported or constrained its use across providers. In this way, data interrogation enabled the research team to build explanations in the form of CMO configurations that explained where NCA data were used to stimulate quality improvement and why.

### Ethics approval

Ethics approval for the study was received from the University of Leeds, School of Healthcare Research Ethics Committee (Approval no. HREC16–044).

## Results

In total 54 participants were recruited across the five study sites; Table 2 provides a summary of the professional roles sampled per Trust. Interview length ranged between 33 and 89 min (mean 54 min).

**Table 2:** Recruitment summary, Role by Trust

Role	Teaching Hospital			DGH		Total
	1	2	3	4	5	
Doctors	4	2	2	4	1	**13**
Nurses	2	7	2	2	2	**15**
Audit support staff	1	2	1	0	0	**4**
Trust Board and Committee Members	2	0	1	1	0	**4**
Quality and safety staff	2	2	1	1	1	**7**
Information staff	3	1	1	0	1	**6**
Other	3	0	1	0	1	**5**
**Total**	**17**	**14**	**9**	**8**	**6**	**54**^**a**^

^a^Includes 14 participants directly involved with MINAP, 12 involved with PICANet, 2 with NACR, 1 with BAUS and 25 with NCAs in general

Below we present our findings, beginning with a description of the types of NCA feedback available to Trusts, before explaining in realist terms how, why and in what contexts this feedback was used by providers and with what impact. These explanations are summarised as CMO configurations in Table 3.

## What NCA feedback is available to providers?

Participants reported that there were three modes through which they interacted with NCA feedback:

1. From public reports produced by NCA suppliers (typically published annually both online and in hard copy);
2. From standardised data reports accessed via NCA supplier websites (available for some audits);
3. From reports produced by staff within clinical services using data obtained from suppliers or extracted from services’ own databases (where these existed).

## Reports produced by NCA suppliers: how, why and in what circumstances are they used?

Participants across all sites reported that a designated NCA lead, typically a consultant, received a copy of the publically-available annual report produced by NCA suppliers. The NCA lead then disseminated a summary of the report to Trust groups with a remit for monitoring care quality. However, the report and summary were reported to receive little response outside the clinical service. For example, after the publication of a PICANet report, a Paediatrician commented:

*‘I'm not invited to the Board meeting to discuss* [the PICANet annual report], *[ … ] there's never any discussion with me directly about it and what it means*.’ *(Paediatrician, Site 1).*

Similarly a Cardiologist (Site 4) noted that in response to their recommendations for change: ‘*absolutely nothing, nothing changes. Why collect the data?’* By way of explanation, the Cardiologist explained that when MINAP was initiated ‘*Cardiology was very high on the political agenda […] and so you could get people to enact the changes and the recommendations’*. However, they believed that the priority accorded to MINAP diminished once the original changes it was designed to promote had been adopted widely across Trusts. This shift in priorities appeared to constrain access to resources when quality improvement opportunities were identified, as another Cardiologist commented:

*‘If you present them* [Trust Board] *with a problem I think they just think let’s not look at that because it might cost us some money.’ (Cardiologist, Site 4).*

In addition, participants explained that data presented in NCA feedback did not always support recommendations for change, as two Information Managers (Site 1) discussed:

Senior Information Manager: *‘There are some national audits where we’ll get a report that’s published within 2017, ‘18, that they call their 2016 audit, that is reporting you the data from 2015/16 patients and if we’re getting that this year, it’s already two years out of date, you know.’*

Information Manager: *‘We’ve already changed our practice by then.’*

In other words, data in the public reports could be up to 2 years old and was not, therefore, perceived as a reliable basis for practice change. Timeliness of data was reported as a significant attribute for use in quality improvement across services. In theory, more timely data could be accessed via supplier websites, for example, PICANet and MINAP have quarterly data upload targets. Even so, some sites were not resourced to meet those targets, as an Information Analyst (Site 3) noted: ‘*We’re a long way off being resourced to do that* [quarterly uploads] *as it stands.’* Better resourced services experienced challenges in accessing timely data also, as an audit clerk in one such service commented:

*‘You can actually wait quite a long time for data to be sent to you* [by the NCA supplier], *by which time you’re like, well, actually I needed it yesterday.’ (PICANet audit clerk, Site 1).*

Equally, clinicians reported constraints on their time to log on to supplier websites to review the standardised data reports available: *‘the reality of our lives in the NHS, is that we don’t have time to do that’ (Urological surgeon site 4)*. Therefore, in some services the primary mode of NCA feedback could be limited to the public report, in which data quality (accuracy and completeness), alongside timeliness, could also constrain its perceived usefulness for stimulating quality improvement. For example, a Cardiologist (Site 5) described that, in a previous year, inappropriate coding of data had resulted in too many cases of myocardial infarction being entered into the database, which:

‘*had a knock-on effect in that some of the other variables, like the number of times the patient was on the heart ward or et cetera were low.*’

Inaccurate coding impacted on the validity of the measures reported, and acted as a constraint on the perceived usefulness of the feedback as a basis for practice change. Data quality was often linked with the data collector’s skills and/or experience, which varied between audits and sites. For example, a surgeon participating in the BAUS audits explained that they trusted the accuracy of their data because: ‘*I know that I’ve written every bit of that data myself’ (Urological surgeon, Site 4).* In comparison to BAUS, data collection for NCAs that reported at service, rather than specific procedural-levels, was supported by a mix of clinical and non-clinical staff. In these contexts, the importance of clinical support for data validation was highlighted. However, even where services were satisfied with the accuracy of their own data, there was apprehension about the quality of other organisations’ data against which their performance was compared.

In summary, participants across Trusts reported interacting with feedback produced by NCA suppliers. However the resources allocated to support audit participation (which impacted data quality and timeliness) and access to resources to enact change in response to this feedback were reported to constrain its use as a tool for stimulating quality improvement. Despite these constraints, however, examples of how and why (the mechanisms) different groups within Trusts interacted with and acted on feedback produced by NCA suppliers, and the circumstances (the contexts) that influenced these interactions, were provided and are discussed below.

### Protecting trust reputation

Trust Boards and their sub-committees monitor performance of services across their organisations. As NCAs report about specific clinical specialities and patient groups, members of these governing groups discussed that their interactions with NCA feedback was typically limited to particular circumstances, as explained by this Trust Board member:

*‘The division of medicine would oversee quality of the stroke service, so there would be the SSNAP audits, the stroke performance, stroke mortality etc., etc. through there, and feed up areas of concern through to the appropriate committee then through to quality committee and if necessary to Trust Board.’ (Trust Board Member, Site 2*)

The participant explained that clinical specialities are responsible for monitoring and improving the quality of their service, but escalate areas of concern to Trust management groups. In these circumstances, participants reported that Trust Boards were more likely to respond to NCA feedback when the clinical service audited was to appear as an outlier in the public report, as described by this Trust Board member:

*‘If we’re going to be an exception for any measure, the national audit team will get in touch with us, and say, you’re going to be an outlier for this particular metric. And, you usually get a short period of time to check your data, and also then, if the data’s correct, for you to write a comment, which they don’t necessarily always commit to publish, but they say they might put a comment on their website for example.’ (Trust Board Member, Site 1)*

Here, rather than the feedback itself, notification from the audit supplier prior to public reporting, prompted a response from the Trust Board. A Paediatrician (Site 1) commented that Trust Boards were particularly responsive to performance measures considered publically sensitive in nature: ‘*they* [the Trust Board] *want to know that we’re not killing more patients because that’s the first thing that the press pick up on’.* Put simply, Trust Boards appeared keen to maintain public confidence in their hospitals’ reputations for providing safe and effective care and acted on NCA feedback where this was brought into question. Where services were identified as outliers in this way, Trust Boards or their sub-committees continued to monitor them, until satisfied that appropriate standards had been met, as described by a Quality Manager (Site 1):

*‘They* [the Trust Board] *want to see the on-going reporting into that group until the point where they say,’ okay, we’re assured about this now*.

In two examples provided by clinicians of such an incident, the subsequent thorough investigations performed by the clinical service, established that data quality and/or patient case mix explained the outlier status. The outcome of these interrogations, therefore, was assurance that the service was performing within expected parameters. Furthermore, in response to being flagged as an outlier, a clinician within an affected service reported monitoring the standardised reports available via the supplier website on a more frequent basis: *‘We had a lot of deaths all together, which started to flag us up […]. It just flagged us up once and that was it, we were on it like a car bonnet, we were just looking at it all the time.’(Consultant, Site 1).*

### Improving performance

In comparison to Trust Boards acting on notification from audit suppliers, a surgeon who participated in the BAUS audits explained how the audit report (produced by the NCA supplier) had stimulated quality improvement. In a previous year the report had highlighted their service as an outlier, in comparison to peer organisations, in relation to patient length of hospital stay. The audit lead discussed the report findings in a clinical governance meeting (as part of standard practice) and in response a number of initiatives were introduced to improve service performance. The surgeon commented that the service changes:

*‘meant that we could get patients out over the weekend, rather than everyone having to wait until the Monday, […] So, yes, it [the NCA report] does have, certainly in our own department, if you are looking like an outlier, we would try hard to address that’. (Urological surgeon, Site 4)*

This surgeon reported that they did not review national comparator data via the NCA supplier website routinely owing to constraints on their time. However, as noted previously, they trusted the accuracy of the data reported, and further commented that they could make judgements about the quality of other organisations’ data based on what was presented. In this context, therefore, the NCA report was trusted as a reliable measure of service performance and national comparison enabled the service to assess where improvement could be made and, where resources allowed, they acted to improve performance.

### Attracting patient referrals

Competition and patient choice have been used as strategies to deliver improvements in the NHS e.g. by offering patients a wider choice of provider and introducing funding systems such as Payment by Results [23]. Therefore, alongside identifying areas for improvement, national comparator data (available in NCA supplier feedback) were also useful to clinical services that wanted to attract patient referrals, as described by a Cardiologist (Site 3):

*‘There are certain centres that are in the capture area of several tertiary centres* [centres that offer specialist services] *and so there is obviously a competition, if you can call it that, for the tertiary centres to capture those patients and so looking at our data and how well we’re performing and how quickly we’re able to offer this service is quite important. Because you want to be able to say to these centres, look, if you refer to us your average wait is a day. If you refer to centre Y your average wait’s a week.’*

In this context of competition, the primary use of NCA feedback was to attract patient referrals to the service; used in this way, however, feedback may stimulate practice change if the service is not performing well in comparison to their peers.

## Reports produced by the clinical service: how, why and in what circumstances are these used?

We found that most frequent interaction with feedback, generated from data collected for participation in NCAs, was reported by staff within clinical services that stored data in local databases, often developed using Microsoft Access or Excel, before upload to the supplier. These databases were maintained (data collected and input) by staff employed to support audit participation and who were able to extract data and customise feedback (produce performance reports), when requested (typically by clinical staff), to meet the needs of their service. Further underpinning use of this data was clinical staff trust in their accuracy; as a clinician in one such service commented:

‘The PICANet data, via [audit clerk], to me is the gold standard of our activity. […] I can rely on the data I get from that’. (Paediatrician, Site 1)

In this service an audit clerk (with over 10 years of experience in the role) worked with the audit lead - who described their service as ‘*obsessed’* with data - to validate the data collected. Similarly, in a cardiology service, an audit clerk and two chest pain nurses worked together to collect and validate MINAP data that were stored locally before upload to the supplier. Therefore, where data were trusted as an accurate reflection of service performance due to the skills, knowledge and experience of staff supporting data collection and where it was easily accessible via local databases, feedback was generated by audit support staff and was reported to be used in the following ways.

### Integrating NCA feedback into routine monitoring processes

In one clinical service, performance reports were produced monthly by the audit coordinator (using data collected as part of PICANet) for review by the clinical team in their clinical governance meetings. The reports consisted of bar charts that displayed service performance in certain measures on a month-by-month basis. The audit lead provided an example of how this feedback had stimulated quality improvement:

*‘We started to look and see a small spike in self-extubations* [unplanned removal of a breathing tube] *of patients and then, so we introduced a monograph where we trained all the nurses in taping and how to check a chest x-ray about tube position. […] then we followed up at the clinical governance meeting, whether or not we had made an impact on the rate of accidental extubations. So, it very easily gives you points to monitor, you know. The data that we collect is very in-depth, it’s very accurate. Because it’s quick and easy to access, it means that we can use it.’ (Paediatrician, Site 1)*

By monitoring the numbers of accidental extubation within the service month-by-month, clinicians quickly identified a rise in unwanted incidents. A practice change was introduced and the impact of that change assessed because the data were ‘*quick and easy to access*’ via the local database. In a similarly resourced cardiology service, a MINAP Assistant (Site 2) reported ‘*I do reports, like monthly and yearly, to show how we’re doing’* and referred to measures of interest:

‘*For the primary PCI patients, there’s a national guideline. We have to…from when they call for an ambulance, to when they get the balloon inflated in their body, we have to do that within 150 minutes. From when they arrive at our door, to when we put the balloon in, that has to be done within 90 minutes. So, they’re our targets*.’

These measures are typically referred to as ‘Call-to-Balloon’ and ‘Door-to-Balloon’ and are captured as part of MINAP (see Table 1). The MINAP assistant explained, whilst they produced the report, it was not ‘*their data*’ and ‘*it’s up to them [*clinical staff*] what they do with them […] I just sort of give the data out and then it’s up to the management and the consultants, really, to improve the service.’* A chest pain nurse, who supported data collection and validation, explained how such reports were used:

*‘We started to notice delays occurring in patients getting to the cath* [catheterisation] *lab in time, what we actually found was that it was because the time to their initial ECG* [electrocardiogram] *was getting longer, and as a result, they then had to put in a new pathway of, if a patient presents with chest pain, they get handed a red card. They then walk to the nearest member of staff and hand it to them and that person has to do an immediate ECG on them, because people that were self-presenting were waiting two or three times as long for a heart trace. So that database* [service database used to store MINAP data] *enabled us to identify that problem, and as a result, a whole new process was put in place to deal with it.’ (Nurse, Site 2)*

Data collected for participation in MINAP, therefore, were used to confirm the delay taking patients to the cath lab where treatment and diagnostics are performed and a service change was instigated to improve performance. The importance of being able to customise reports in this way was highlighted by a Cardiologist working within a service not resourced for this purpose:

‘*The only analysis we get is that that’s done by MINAP, and it might be that if we’re looking at the data from our own perspective that we can pick out subtler changes that, you know, it might be that there’s an hour delay in somebody getting an ECG in A&E – that’s not going to be picked up in MINAP, right, whereas it’s something that we might be able to effect change very quickly and that would be a quality improvement and you could do that, you know, you don’t need that to go to a national level to tell you, do you? In fact it’d be missed at national level.’ (Cardiologist, Site 4)*

This service was not resourced to generate reports from local or supplier held data, therefore they were reliant on feedback produced by the supplier – notably the annual report - that did not offer a level of detail that enabled them to ‘pick out subtler changes’ where quality improvement might be delivered.

Use of feedback in this way was attributed to professionalism; clinicians’ motivation to ensure that the care delivered to patients is safe and effective. For example, a Paediatrician (Site 1) commented: ‘*if there’s something I can change I should be changing it […] as soon as I am aware*’. Where resourced, feedback could be generated routinely, or as requested, for this purpose. However, some measures reviewed in this way were attached to incentives also, as discussed below.

### Obtaining incentives

A number of PICANet measures, including accidental extubation (discussed above), are part of the CQUINs funding initiative. As such they are reported to NHS England on a quarterly basis by all PICUs and their performance impacts service funding. In MINAP, the measure Door-to-Angiography is part of the BPT, whereby services are financially rewarded for achieving a target, as a MINAP Assistant (Site 2) discussed:

*‘For the financial year 2017/2018, […] we just managed to scrape it. But we’re hoping to do better this year because […] we’re hoping to get nurses on a weekend, just to try and make sure that all the patients are having the angio within 72 hours.’*

This service aimed to achieve the tariff and made use of data collected as part of MINAP in order to monitor performance against the target. This example highlights how multiple mechanisms may underpin use of NCA feedback and provide opportunities to stimulate quality improvement. Furthermore, in the context of the NACR audit, incentives that operated in a different way were identified. A Nurse (Site 4) discussed accreditation from NACR, which requires meeting certain standards of care, as a driver for engagement with NCA feedback:

*‘It is, definitely* [a driver for use of NCA data] *[…] Yeah, put the service on the map which will then look good for patients looking online, you know, this is a certified service and the certification process, I think it lasts three years before you have to reapply.’*

Certification status was published for the first time in the NACR 2018 report and indicates how initiatives additional to the feedback itself drive its use. Interestingly, use of NACR feedback was reported in a site where resources constrained routine use of MINAP data. One reason for this was that the cardiac rehabilitation service used an Excel spreadsheet to record their data locally, as they were unable to access reports in a more timely way via the supplier. The nurse explained: ‘*The computer systems that we were told would be able to pull off all these reports don’t*, *we had to come up with a live system*.’ This highlights that clinical services within the same Trust can have different resources and opportunities to interact with NCA feedback.

### Developing professionals

Trainees (doctors and nurses) complete projects, including internal audits, as part of their professional development. We found that trainees use NCAs if they provide information that are useful for specific projects. Data to support the project could be accessed via suppliers, but engagement appeared strongest in the services where NCA data could be easily accessed via service databases and where there were staff available to support the processes involved in data extraction. Depending on the project, the findings could be used to inform service improvement, as indicated by this clinician:

‘We’ve used the PICANet database and we looked at the […] burden of prematurity on the PICU, which has got implications for long term finance, you know. If we’re producing a lot of neonates, they put a big burden on PICU services in the future. […] it can change what your perceptions are, because you’ve got accurate data. So, you know, if you actually look at something in particular, you know, it can actually change your outcome and change where you’re spending money.’ (Paediatrician, Site 1)

#### Context+Mechanism = Outcome configurations

In summary, we found that interactions with NCA feedback were underpinned by a number of mechanisms, but they varied in terms of mode of feedback, frequency and impact. To summarise our findings in realist terms we configured a programme theory in the form of a series of CMO configurations, see Table 3.

**Table 3:** How, why and in what circumstances is NCA feedback used to stimulate quality improvement

Context		Mechanism		Impact / Outcome
In what circumstances	For whom	NCA Resource	Provider Response	
NHS Trusts operate in a context of competition, public choice and funding initiatives designed to stimulate quality improvement.	Trust Boards and their sub-committees that have oversight of clinical services across their organisation.	Trust Boards are notified via the NCA supplier if a clinical service is to appear as an outlier in the publically available annual report.	**Reputation** The Trust Board acts to preserve Trust reputation for providing safe and effective care**,** particularly in response to measures considered publically sensitive in nature.	Data interrogation to establish cause of the outlier status, which may lead to more frequent monitoring of the clinical service for assurance.
Professional groups within Trusts have different improvement priorities, and power to support service changes.	Clinicians who trust that the feedback is accurate as they upload data to the NCA supplier directly, but do not monitor measures via the NCA supplier website routinely due to constraints on their time.	The public report produced by NCA suppliers offers national benchmarks against which to compare service performance.	**Professionalism**: Clinicians incorporate the NCA report into the service’s clinical governance processes, to assess service performance and where improvements can be made.	Supplier feedback highlights if the service is an outlier in comparison to peer organisations. The clinical service makes changes to improve their performance, where resources allow.
	Tertiary centres that compete with other organisations for patient referrals from district hospitals.	The public report produced by the supplier enables services to benchmark their performance against peer organisations in target-based measures.	**Competition:** The clinical service uses the feedback to evidence competitive performance to feeder services.	Feeder services may choose to refer more patients to the centre. Clinical teams may act to improve performance to attract patient referrals.
	Clinical services resourced to collect accurate and timely data + to maintain local databases where NCA data is stored prior to upload to the NCA supplier	Audit support staff customise feedback using local data i.e. without national comparators.	Measures considered important for ***professionalism*** and/or to obtain ***incentives*** (financial or accreditation) are integrated into the service’s monitoring processes.	Clinical staff can quickly identify rises in unwanted incidents or delays in treatment times, introduce change to improve performance, where resources allow, and asses the impact of the change.
	Junior doctors and nurses are expected to complete projects as part of their placement within the clinical service.	NCAs (via supplier or local databases) offer data that can be used to address trainees’ research questions.	**Professional development –** with support from audit support staff, trainees extract raw data for their projects. Projects provide learning about the service that may highlight how it can be improved.	Knowledge/lessons from research projects might be used to inform service delivery.

## Discussion

In this paper we have reported the development of the first programme theory constructed to help understand variation in the use and impact of NCA feedback. Using realist evaluation as a study framework, we identified a number of mechanisms that explained why different groups within provider organisations engage with NCA feedback; these were labelled reputation, professionalism, competition, incentives and professional development (summarised in Table 3). These mechanisms resonate with previous studies: for example, public reporting has been found to engage mechanisms similar to those labelled here as reputation and professionalism [24]. Guided by realist evaluation, however, we interrogated the data to understand what contextual supports and constraints shaped interactions with NCA feedback and were particularly interested to understand where feedback was used to stimulate quality improvement.

NCAs may be considered an example of a ‘boundary object’, which superficially looks to align the interests of different stakeholders [25, 26]. However, we found that interactions with NCA feedback differ amongst provider groups that have different responsibilities and priorities for monitoring and improving services. Trust Boards, for example, engaged with NCA feedback to a limited extent and in particular circumstances e.g. if it brought the Trust’s reputation into question. Furthermore, staff within clinical services reported that access to provider resources to support change, based on NCA feedback, could be constrained, particularly if those services appeared to be performing well in metrics of public interest. Therefore, interactions with NCA feedback can be limited to assurance (compliance that performance is in line with certain standards) rather than contributing to a continued effort to improve outcomes, which typically characterises quality improvement [27, 28]. This finding has particular implications for well-established audits, such as MINAP and PICANet, where more substantial change efforts in response to NCA feedback have been successfully established historically [2, 5].

Whilst power differentials between clinical and governing groups may constrain access to provider resources to support change, challenges accessing supplier held data, and concerns about data quality and timeliness constrained the perceived usefulness of this feedback by staff within clinical services. Most proactive and routine use of feedback, produced using data collected for participation in NCAs, was reported in clinical services resourced to generate reports from local databases, where data were stored before upload to NCA suppliers. Underpinning use of these data was clinical staff trust that it was an accurate and timely reflection of service performance, due to the skills, knowledge and experience of staff maintaining the local databases. In these circumstances, feedback was generated that enabled clinical services to monitor performance month-by-month or against national targets (where available), but without national comparator data.

### Strengths and limitations

Previous research has highlighted data quality and relevance as potential constraints on use of feedback [3, 7, 29] . The findings of this study support this work, and we have configured these constraints, and others, as contextual influences on the mechanisms through which NCA feedback might stimulate quality improvement. To help explain variation in use of feedback, our sampling strategy captured variation in NCAs, healthcare providers, and healthcare professionals. In this way, we were able to explore how different provider organisations, services and professional groups interacted with NCA feedback and for what purpose. The resulting CMO configurations provide insight into how and why NCA feedback is used to stimulate quality improvement in some contexts but not in others.

The study was limited, as we focused our attention on two well-established audits. Therefore we were unable to capture their initial impact, where examples of if/ how they worked to align the priorities of different provider groups to stimulate quality improvement may have been reported. The CMOs developed are also ‘first order’ programme theories and remain to be empirically tested [18]. For example, incentivising performance was found to engage providers in the use of feedback in this study, but in other settings potential adverse effects on measures not incentivised have been identified (31). We asked our participants specifically how and why NCAs were used, as opposed to exploring potential adverse impacts. As realist evaluation advocates an iterative process of theory testing and refinement, questions about adverse impacts could be explored as part of theory testing to build knowledge about NCA feedback cumulatively.

## Conclusions

The CMOs developed indicate that there are a number of mechanisms that underpin provider use of NCA feedback, but there is variation in the mode, frequency and impact, depending on the circumstances. Feedback produced by NCA suppliers, which included national comparator data, was used in a limited capacity. A number of constraints were identified that impacted the perceived usefulness of this mode of feedback as a tool for stimulating quality improvement. Most proactive and routine use of feedback, providing opportunities to stimulate quality improvement, was identified within clinical services, where data were easily accessible via locally managed databases, and trusted as an accurate reflection of service performance. These attributes in turn were dependent on the resources allocated to support audit participation and data use, which varied across providers.

## Data Availability

In line with the ethics approval, the data will be kept until June 2030 and can be accessed by other researchers during this time, subject to the necessary ethical approvals being obtained. Requests for access to this data should be addressed to the corresponding author.

## Abbreviations

BAUS: British Association of Urological Surgeons
BPT: Best Practice Tariff
CMO: Context Mechanism Outcome
CQUIN: Commissioning for Quality and Innovation
HQIP: Health Care Quality Improvement Programme
MINAP: Myocardial Ischaemia National Audit Project
NACR: National Audit of Cardiac Rehabilitation
NCA: National Clinical Audit
NHS: National Health Service
PICANet: Paediatric Intensive Care Audit Network
UK: United Kingdom
